# Association of the Inflammatory Burden Index With Increased Mortality Among Cancer Patients: Insights From the NHANES Study

**DOI:** 10.1002/iid3.70067

**Published:** 2024-12-06

**Authors:** Xiuxiu Qiu, Yiyi Zhang, Yingjie Zhu, Ming Yang, Li Tao

**Affiliations:** ^1^ Department of Oncology Longhua Hospital, Shanghai University of Traditional Chinese Medicine Shanghai China; ^2^ Department of Intensive Care Unit Longhua Hospital, Shanghai University of Traditional Chinese Medicine Shanghai China; ^3^ Department of Good Clinical Practice Longhua Hospital, Shanghai University of Traditional Chinese Medicine Shanghai China

**Keywords:** cancer patients, cardiovascular disease mortality, inflammatory burden index, mortality rates, NHANES

## Abstract

**Background:**

The systemic inflammatory response significantly influences the progression and prognosis of various cancers. The novel Inflammatory Burden Index (IBI) was recently introduced as a biomarker to gauge systemic inflammation and evaluate cancer patient prognosis. However, studies investigating the relationship between IBI and mortality rates in cancer patients remain limited.

**Methods:**

This study analyzed data from 2748 cancer patients enrolled in the National Health and Nutrition Examination Surveys between 1999 and 2018. We used weighted Cox regression analysis and restricted cubic spline models to examine the relationship between the IBI and mortality due to all causes, cardiovascular disease (CVD), and cancer. Furthermore, we employed Kaplan‐Meier survival curves, subgroup analyses, and receiver operating characteristic curves to elaborate on these associations.

**Results:**

Over a median follow‐up period of 112 months, the cohort experienced 1067 deaths, including 320 from cancer, 239 attributable to heart disease, and 508 from various other causes. The Kaplan‐Meier curve indicated that individuals in the higher quartiles of the IBI faced significantly increased mortality risks compared to those in lower quartiles. Analyses using weighted Cox proportional hazards models demonstrated that subjects in the top IBI quartile were at a substantially higher risk for all‐cause mortality (Hazard Ratio [HR] 2.09, 95% Confidence Interval [CI] 1.67–2.62, *p* < 0.001), CVD mortality (HR = 1.95, 95% CI= 1.18–3.23, *p* = 0.010), and cancer mortality (HR = 2.06, 95% CI = 1.31–3.26, *p* = 0.002). Furthermore, stratification and interaction analyses affirmed the uniformity of these initial findings. The areas under the curve for the 3‐, 5‐, and 10‐year survival predictions for all‐cause mortality were 0.62, 0.62, and 0.67, respectively; for cardiovascular mortality, they were 0.64, 0.64, and 0.70; and for cancer mortality, they were 0.62, 0.77, and 0.70.

**Conclusion:**

In cancer patients, higher IBI levels significantly correlate with increased mortality from all causes, CVD, and cancer‐specific deaths. This index could possess considerable diagnostic and prognostic importance, possibly acting as a new biomarker for evaluating outcomes in cancer patients.

## Graphical abstract

Our research employs data from the National Health and Nutrition Examination Survey spanning from 1999 to 2018. The study reveals that higher Inflammatory Burden Index levels significantly correlate with increased mortality from all causes, cardiovascular disease, and cancer‐specific deaths in cancer patients.

## 1 Introduction

In recent years, the prevalence and mortality rates of cancer have presented major challenges to global public health. Global statistics from 2022 show nearly 20 million new cancer cases and approximately 10 million deaths. Projections based on demographic trends suggest that by 2050, the annual incidence of cancer will rise to 35 million, representing a 77% increase compared to 2022 levels [1]. Systemic inflammation, a hallmark of host‐tumor interactions in cancer patients, has been shown to critically influence tumor initiation, progression, metastasis, and resistance to therapy [2]. A higher inflammatory burden in these patients typically correlates with worse prognostic outcomes [3]. Moreover, a growing body of research underscores the prognostic significance of inflammatory biomarkers in estimating the overall survival (OS) of cancer patients [4].

Inflammatory markers, such as the neutrophil‐lymphocyte ratio (NLR) [5], the platelet‐to‐lymphocyte ratio (PLR) [6], and the lymphocyte‐to‐C‐reactive protein (CRP) ratio [7], have been established as independent prognostic indicators across various tumors. However, these indicators fall short of offering a holistic assessment of a patient's inflammatory burden, which restricts their diagnostic and prognostic effectiveness. In the evolving landscape of inflammatory markers, the newly introduced Inflammatory Burden Index (IBI) has attracted substantial interest from researchers for its potential to predict cancer outcomes [8] accurately.

Comprehending the link between the IBI and cancer mortality could improve prognostic precision for cancer patients. Recognizing groups at higher risk of mortality enables the implementation of focused interventions aimed at reducing inflammation and enhancing patient outcomes. Additionally, the IBI may prove to be an essential tool for assessing the effectiveness of anti‐inflammatory treatments in both clinical and research environments.

Taking these factors into account, this research employs data from the NHANES spanning from 1999 to 2018. The main goal is to investigate the association between the IBI and mortality due to all causes, cardiovascular disease (CVD), and cancer. This analysis seeks to validate the effectiveness of the IBI in diagnosing and prognosing cancer.

## 2 Materials and Methods

### 2.1 Data Sources

Data utilized in this investigation originated from the National Health and Nutrition Examination Survey (NHANES, https://www.cdc.gov/nchs/nhanes/), a comprehensive survey gathering data on the nutritional and health status of civilians outside of institutional settings across the United States. This program is administered by the National Center for Health Statistics (NCHS), a component of the Centers for Disease Control and Prevention (CDC). The NCHS Ethics Review Board has sanctioned all NHANES protocols, and written informed consent was obtained from each participant's legal guardians or next of kin. Moreover, compliance with the STROBE guidelines is maintained in this study to uphold strict reporting standards for observational research.

### 2.2 Study Population

**Figure 1:**
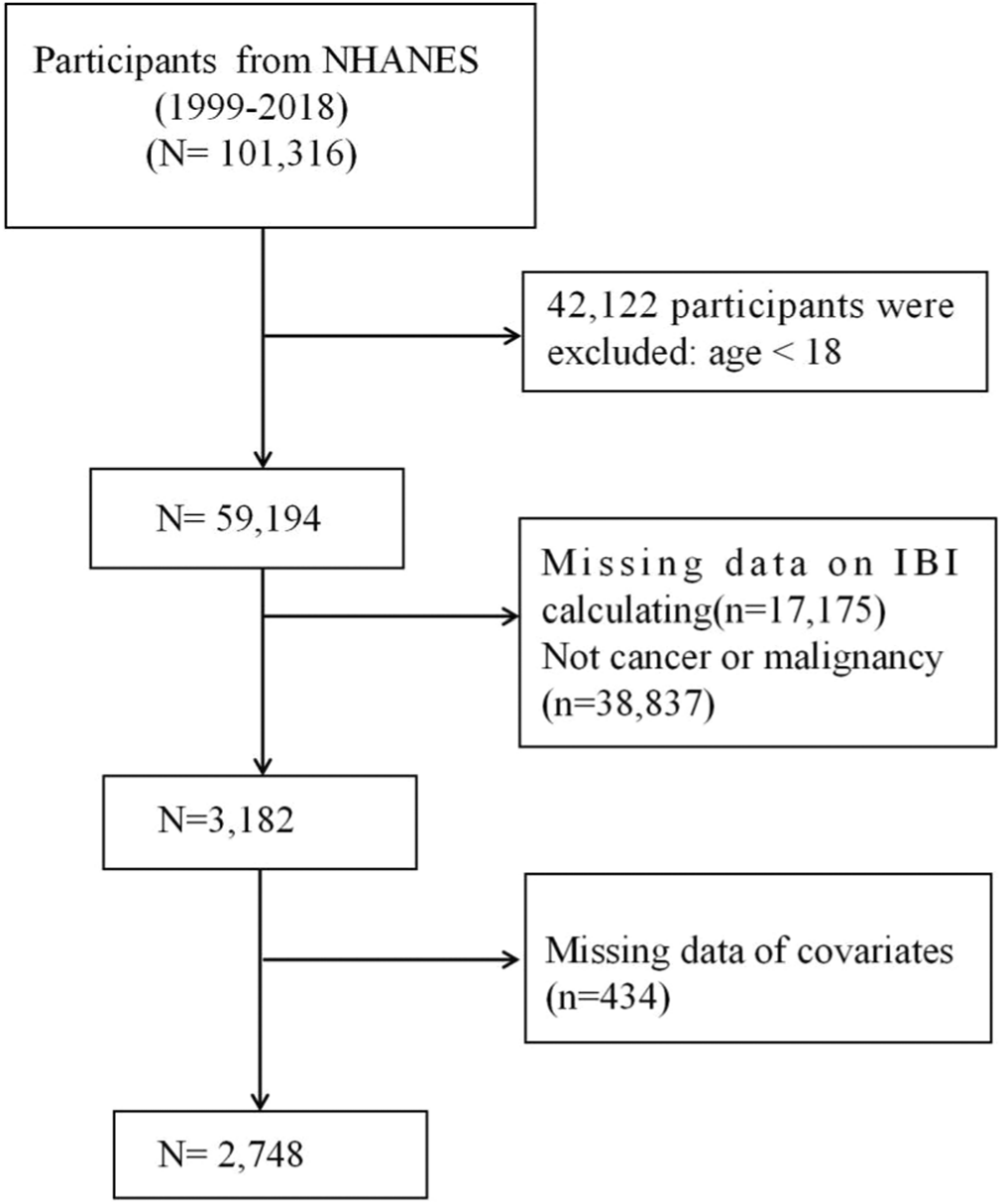
Flowchart of the participant's selection from NHANES 1999–2018.

From 1999 to 2018, a total of 101,316 participants were initially considered for the study. We first excluded individuals under 18 years of age (*n* = 42,122). Subsequently, participants lacking the necessary data to compute the IBI, particularly absent values for C‐reactive protein, neutrophil count, and lymphocyte count, were excluded (*n* = 17,175). The determination of cancer status was based on self‐reported diagnoses. The question posed to participants was, “Have you ever been informed by a doctor or another health professional that you have cancer or any type of malignancy?” Those who answered “yes” were classified as cancer patients. We further excluded participants who either had no history of cancer (*n* = 38,837) or lacked data on relevant covariates (*n* = 434). As a result, the final group analyzed in our study consisted of 2748 individuals who had survived cancer (Figure 1).

## 3 Calculation of IBI

The IBI was determined by the formula: IBI = CRP ×(neutrophil count/lymphocyte count), which was derived from laboratory data to calculate systemic inflammatory markers [8]. For the analysis, IBI scores were divided into quartiles. The first quartile indicates a low level of inflammation, and the fourth quartile indicates a high level of inflammation. The ranges are defined as follows: Quartile 1: IBI < 0.289; Quartile 2: 0.289 < IBI < 0.832; Quartile 3: 0.832 < IBI < 2.641; Quartile 4: IBI > 2.641.

### 3.1 Assessment of Mortality

The status of mortality was confirmed by linking NHANES data with records from the National Death Index (NDI). Further details on this linkage are available at the CDC website: https://www.cdc.gov/nchs/data-linkage/mortality-public.htm.Variables in the analysis included mortality status and duration of follow‐up, categorizing outcomes into two distinct groups: survival and death. Cancer mortality refers to the probability of death from various malignant tumors, while noncancer mortality denotes the probability of dying from causes other than cancer. Cardiovascular Disease Mortality is characterized by the likelihood of dying from heart and blood vessel diseases. Mortality status and causes of death were ascertained using records from the NDI, with data accessible up to 31 December 2019. The causes of death among participants were categorized based on the 10th revision of the International Classification of Diseases (ICD‐10), which encompasses cardiovascular diseases (codes I00–I09, I11, I13, I20–I51) and cancers (codes C00–C97).

### 3.2 Assessment of Covariates

The study evaluated continuous variables including age and the family income‐to‐poverty ratio (PIR). Additionally, it considered categorical variables like gender (male/female), ethnicity (Hispanic, non‐Hispanic White, non‐Hispanic Black, and others), levels of education (less than high school/high school or higher), and marital status (Married/cohabitant, Separated/divorced, Widowed, Never married). Body Mass Index (BMI) was determined by dividing the weight in kilograms (kg) by the square of the height in meters (m²). From these calculations, BMI was classified into three categories: underweight/normal ( < 25.0 kg/m²), overweight (25.0–29.9 kg/m²), and obese ( ≥ 30.0 kg/m²) [9]. Smoking status was classified as never smoker (smoked fewer than 100 cigarettes in their lifetime), former smoker (smoked more than 100 cigarettes but has quit recently), and current smoker. Drinking status was classified into five categories based on responses from the past year's questionnaire: never (consumed fewer than 12 drinks in a lifetime), mild (up to 2 drinks daily for males, up to 1 drink daily for females), moderate (between 2 and 3 drinks daily for males, between 1 and 2 drinks daily for females, or 2–4 days per month of binge drinking), heavy (4 or more drinks daily for males, 3 or more drinks daily for females, or 5 or more days per month of binge drinking), and former (no drinking in the past year but had consumed at least 12 drinks in any previous year or over a lifetime). Chronic diseases such as diabetes, hypertension, coronary heart disease (CHD), chronic kidney disease (CKD) and hyperlipidemia were assessed in the study. Diabetes was categorized into diabetes mellitus (DM), impaired fasting glycemia (IFG), and impaired glucose tolerance (IGT). The diagnoses of hypertension, CHD, and hyperlipidemia were determined based on the patient's self‐report [10].

### 3.3 Statistical Analysis

Demographic data, medical records, and laboratory results were collected from NHANES. To accurately mirror the U.S. demographic profile, our study design included appropriate sample weights, stratification, and clustering in our analysis. For continuous variables, they are displayed as the mean ± standard deviation, while categorical variables are shown as numbers (percentages). Differences between groups were assessed using the survey‐weighted Chi‐square test, which compares observed frequencies to expected frequencies under the null hypothesis of no association between variables. Hazard ratios (HRs) and 95% confidence intervals (CIs) were estimated using weighted multivariable Cox proportional hazards regression models to assess the relationship between the IBI and mortality in cancer patients. Three distinct models were developed: Model 1 remained unadjusted; Model 2 incorporated adjustments for age, gender, and ethnicity; Model 3 further included adjustments for BMI, education level, PIR, smoking and drinking status, as well as a personal history of hypertension, CKD, diabetes, hyperlipidemia, and CHD. Subsequently, the Kaplan‐Meier method was used to assess the survival probabilities of cancer patients across various quartile levels of the IBI. Furthermore, we utilized restricted cubic splines (RCS) with adjustments for covariates to demonstrate the linear or nonlinear relationships between the IBI and mortality. After this, subgroup analyses were performed to address potential confounding factors. The effects observed in various subgroups and the significance of interactions were determined and depicted through forest plots. Additionally, the “timeROC” package was employed to evaluate the precision of the IBI in forecasting survival outcomes at different time points. Two‐tailed P‐values less than 0.05 were considered statistically significant, reflecting the exploratory nature of the study and accounting for potential effects in both directions. All data analyses were conducted using R software version 4.3.2.

## 4 Results

### 4.1 Baseline Characteristics

Table 1 presents the characteristics of the study population, which encompassed 2748 cancer patients aged 18 and older, corresponding to a weighted population of 11,895,824 individuals. The weighted mean age for the study participants was 61.95 years (standard error = 0.37), and females accounted for 57.94% of the population. The participants were grouped into four quartiles based on the IBI: Quartile 1 (*N* = 686), Quartile 2 (*N* = 688), Quartile 3 (*N* = 686), and Quartile 4 (*N* = 688). Of the participants, 2027 (88.53%) were classified as non‐Hispanic White. Typically, participants with elevated IBI were older, primarily of White ethnicity, and had a higher incidence of hypertension and hyperlipidemia, Notably, this group had a lower incidence of CKD and diabetes. Furthermore, those with elevated IBI levels tended to exhibit obesity.

**Table 1:** Baseline information for participants grouped according to IBI level.

Variable	Total	Quartile of IBI				*p* value
		Quartile 1	Quartile 2	Quartile 3	Quartile 4	
***N*, %**	2748	686	688	686	688	
**Age**	61.95 ± 0.37	59.35 ± 0.68	62.76 ± 0.70	62.52 ± 0.73	63.23 ± 0.77	< 0.001
**Gender, %**						0.304
Female	1420 (57.94)	342 (56.36)	350 (55.86)	379 (61.65)	349 (57.90)	
Male	1328 (42.06)	344 (43.64)	338 (44.14)	307 (38.35)	339 (42.10)	
**Ethnicity, %**						0.014
Mexican American	197 (1.93)	41 (1.52)	43 (1.44)	62 (2.48)	51 (2.25)	
Non‐Hispanic White	2027 (88.53)	545 (91.23)	530 (91.05)	483 (85.02)	469 (86.96)	
Non‐Hispanic Black	324 (4.59)	65 (3.51)	79 (4.29)	91 (5.67)	89 (4.91)	
Other	200 (4.95)	35 (3.75)	36 (3.22)	50 (6.83)	79 (5.88)	
**Education level, %**						0.222
High school or above	2470 (94.84)	617 (94.81)	611 (93.83)	616 (94.52)	626 (96.02)	
Below high school	278 (5.16)	69 (5.19)	77 (6.17)	70 (5.48)	62 (3.98)	
**PIR**	3.30 ± 0.05	3.55 ± 0.08	3.24 ± 0.07	3.17 ± 0.09	3.24 ± 0.09	0.001
**BMI**						< 0.001
< 25.0	798 (30.20)	293 (46.52)	206 (30.92)	170 (28.27)	129 (15.82)	
25.0–29.9	990 (34.47)	257 (35.69)	273 (37.53)	224 (30.53)	236 (34.23)	
≥ 30.0	960 (35.33)	136 (17.79)	209 (31.55)	292 (41.19)	323 (49.95)	
**Marital status, %**						0.123
Married/cohabitant	1719 (67.10)	459 (71.63)	442 (67.58)	413 (64.22)	405 (64.97)	
Separated/divorced	421 (14.27)	87 (11.38)	88 (12.51)	123 (17.27)	123 (15.85)	
Widowed	470 (13.87)	103 (11.72)	128 (15.69)	122 (14.78)	117 (13.54)	
Never married	138 (4.75)	37 (5.27)	30 (4.22)	28 (3.73)	43 (5.63)	
**Drinking status, %**						0.065
Former	680 (20.33)	154 (19.07)	182 (20.94)	188 (23.92)	156 (17.78)	
Heavy	262 (11.00)	68 (11.45)	66 (11.12)	54 (8.66)	74 (12.55)	
Mild	1141 (43.52)	309 (45.40)	275 (41.03)	263 (41.99)	294 (45.24)	
Moderate	314 (14.82)	76 (14.21)	87 (17.34)	69 (11.99)	82 (15.79)	
Never	351 (10.33)	79 (9.87)	78 (9.57)	112 (13.43)	82 (8.63)	
**Smoking status, %**						0.078
Former	1145 (39.50)	276 (37.78)	293 (40.78)	279 (36.84)	297 (42.39)	
Never	1184 (44.25)	323 (48.23)	276 (39.55)	306 (45.79)	279 (43.12)	
Current	419 (16.25)	87 (13.99)	119 (19.66)	101 (17.38)	112 (14.49)	
**Hypertension, %**						< 0.001
No	996 (43.36)	315 (54.35)	233 (40.15)	230 (41.37)	218 (37.44)	
Yes	1752 (56.64)	371 (45.65)	455 (59.85)	456 (58.63)	470 (62.56)	
**Diabetes, %**						< 0.001
DM	674 (20.22)	136 (15.21)	136 (15.73)	192 (21.94)	210 (27.27)	
IFG	164 (6.29)	29 (3.56)	30 (3.81)	49 (9.23)	56 (8.36)	
IGT	100 (3.10)	17 (2.05)	30 (3.94)	30 (3.45)	23 (3.06)	
No	1810 (70.40)	504 (79.18)	492 (76.53)	415 (65.38)	399 (61.31)	
**CKD, %**						< 0.001
No	1827 (73.92)	519 (82.23)	453 (72.82)	444 (72.22)	411 (68.50)	
Yes	921 (26.08)	167 (17.77)	235 (27.18)	242 (27.78)	277 (31.50)	
**CHD, %**						0.056
No	2462 (91.69)	635 (93.83)	622 (92.48)	607 (91.46)	598 (89.20)	
Yes	286 (8.31)	51 (6.17)	66 (7.52)	79 (8.54)	90 (10.80)	
**Hyperlipidemia, %**						< 0.001
No	520 (19.31)	160 (26.47)	115 (16.93)	124 (17.20)	121 (16.45)	
Yes	2228 (80.69)	526 (73.53)	573 (83.07)	562 (82.80)	567 (83.55)	
**All‐cause death**						< 0.001
No	1681 (71.58)	429 (74.33)	380 (65.35)	398 (67.75)	474 (77.70)	
Yes	1067 (28.42)	257 (25.67)	308 (34.65)	288 (32.25)	214 (22.30)	
**CVD related death**						0.131
No	1681 (91.84)	429 (92.33)	380 (89.91)	398 (90.83)	474 (93.63)	
Yes	239 (8.16)	61 (7.67)	64 (10.09)	60 (9.17)	54 (6.37)	
**Cancer specific death**						0.031
No	1681 (89.52)	429 (91.71)	380 (86.01)	398 (88.60)	474 (90.97)	
Yes	320 (10.48)	67 (8.29)	95 (13.99)	82 (11.40)	76 (9.03)	

*Note:* Continuous variables are presented as the mean ± standard deviation, while categorical variables are shown as *n* (%). A *p* < 0.05 indicates statistical significance.

Abbreviations: BMI, Body Mass Index; CHD, coronary heart disease; CKD, chronic kidney disease; CVD, cardiovascular disease; PIR, poverty income ratio (the ratio of family income to poverty).

### 4.2 Association of IBI With All‐Cause, CVD, and Cancer‐Specific Mortality

Three distinct models were constructed to assess the independent functionality of the IBI as well as its association with mortality in cancer patients (Table 2). To explore the connection between IBI and mortality, multivariate weighted Cox regression analyses were utilized, making precise adjustments for potential confounders. The results revealed that the IBI functioned as an independent prognostic factor for cancer patients. It was demonstrated through multivariate Cox regression analyses that for each unit elevation in the continuous IBI measure, there was a corresponding 1% rise in the risk of all‐cause mortality, a 1.01% enhancement in the risk of CVD mortality, and a 1% increase in cancer mortality.

**Table 2:** Association of IBI with all‐cause, CVD, and cancer mortality among U.S. cancer patients from the NHANES 1999–2018 cohort.

All‐cause	Model 1		Model 2		Model 3	
Character	HR (95% CI)	*p*	HR (95% CI)	*p*	HR (95% CI)	*p*
IBI (Continuous)	1.01 (1.01, 1.01)	< 0.001	1.01 (1.01, 1.01)	< 0.001	1.00 (1.00, 1.01)	< 0.001
**IBI (Category)**						
Q1	Reference	—	Reference	—	Reference	—
Q2	1.51 (1.24, 1.83)	< 0.001	1.31 (1.09, 1.58)	0.004	1.25 (1.03, 1.53)	0.030
Q3	1.80 (1.40, 2.33)	< 0.001	1.58 (1.27, 1.96)	< 0.001	1.43 (1.13, 1.81)	0.003
Q4	2.52 (2.04, 3.12)	< 0.001	2.35 (1.92, 2.87)	< 0.001	2.09 (1.67, 2.62)	< 0.001
*p* for trend	< 0.001	< 0.001	< 0.001			

**CVD**	**Model 1**		**Model 2**		**Model 3**	
Character	**HR (95% CI)**	***p***	**HR (95% CI)**	***p***	**HR (95% CI)**	***p***
IBI (Continuous)	1.01 (1.01, 1.01)	< 0.001	1.01 (1.01, 1.01)	< 0.001	1.01(1.00,1.01)	< 0.001
**IBI (Category)**						
Q1	Reference	***—***	Reference	***—***	Reference	***—***
Q2	1.43 (0.97, 2.11)	0.070	1.28 (0.86, 1.92)	0.230	0.99 (0.61, 1.61)	0.970
Q3	1.78 (1.17, 2.73)	0.010	1.54 (1.09, 2.17)	0.010	1.12 (0.74, 1.69)	0.580
Q4	2.97 (1.89, 4.68)	< 0.001	2.64 (1.65, 4.24)	< 0.001	1.95 (1.18, 3.23)	0.010
*p* for trend	< 0.001	< 0.001	0.020			

**Cancer**	**Model 1**		**Model 2**		**Model 3**	
Character	**HR (95% CI)**	***p***	**HR (95% CI)**	***p***	**HR (95% CI)**	***p***
IBI (Continuous)	1.01 (1.01, 1.01)	< 0.001	1.01 (1.01, 1.01)	< 0.001	1.00 (1.00, 1.01)	< 0.010
**IBI (Category)**						
Q1	Reference	—	Reference	—	Reference	—
Q2	1.85 (1.25, 2.74)	0.002	1.64 (1.13, 2.39)	0.001	1.42 (0.93, 2.16)	0.100
Q3	1.99 (1.30, 3.05)	0.001	1.92 (1.25, 2.94)	0.003	1.81 (1.20, 2.73)	0.005
Q4	2.98 (1.88, 4.72)	< 0.001	2.62 (1.65, 4.15)	< 0.001	2.06 (1.31, 3.26)	0.002
*p* for trend	< 0.001	< 0.001	< 0.001			

*Note:* Model 1: Unadjusted; Model 2: Adjusted for age, ethnicity, and gender; Model 3: Further adjusted for BMI, marital status, education level, drinking status, smoking status, PIR, hypertension, diabetes mellitus (DM), CHD, CKD, hyperlipidemia, and cancer type.

Participants were divided into four groups according to the IBI quartiles, with the first quartile used as the reference category. Model 1 incorporated no covariate adjustments. Model 2 adjusted for factors such as age, gender, and ethnicity. Model 3 included additional adjustments for BMI, marital status, education level, drinking and smoking habits, PIR, hypertension, diabetes, CHD, CKD, DM, hyperlipidemia, and cancer type.

In comparison to the first quartile of the IBI, the HRs adjusted for various factors, with 95% CIs for mortality from all causes, CVD, and cancer were 2.09 (1.67, 2.62), 1.95 (1.18, 3.23), and 2.06 (1.31, 3.26), respectively, in model 3. The *p* values for trends, which < 0.001 across all models, consistently demonstrate an increase in the risk of mortality from all causes, CVD, and cancer as IBI escalates.

### 4.3 Survival Analysis of IBI for Mortality Risk

Over the median follow‐up period of 111 months, there were 1067 deaths, including 320 from cancer, 239 from heart disease, and 508 from various other causes. A survival analysis was conducted to evaluate the differences in survival among cancers with varying degrees of inflammatory burden.

**Figure 2:**
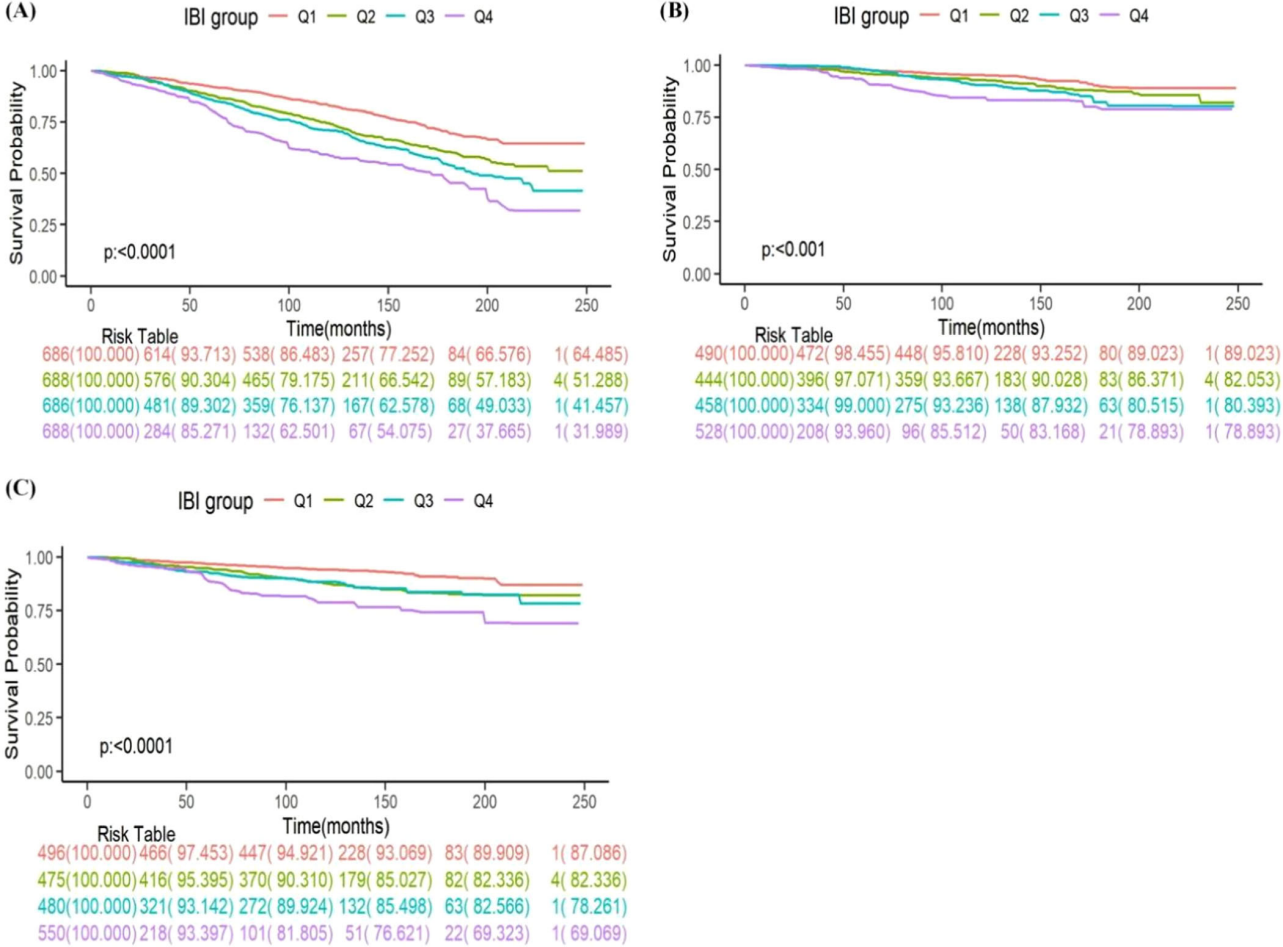
The Kaplan‐Meier curve and risk table for survival probabilities from (A) all‐cause mortality, (B) CVD mortality, and (C) cancer‐specific mortality of cancer patients. The Kaplan‐Meier curves divide the population into four groups (Q1, Q2, Q3, Q4) based on the quartiles of IBI, and statistical analysis is performed using the log‐rank test.

Figure 2 illustrates the Kaplan‐Meier survival curves for all participants, categorized by the quartiles of the IBI. Participants in the highest quartile (Q4) exhibited a significantly increased risk of death from all causes, CVD, and cancer‐specific(log‐rank test, all *p* < 0.001). Those with a higher inflammatory burden experienced poorer outcomes compared to those with a lower burden.

### 4.4 Nonlinear Relationship Between IBI and Mortality

**Figure 3:**
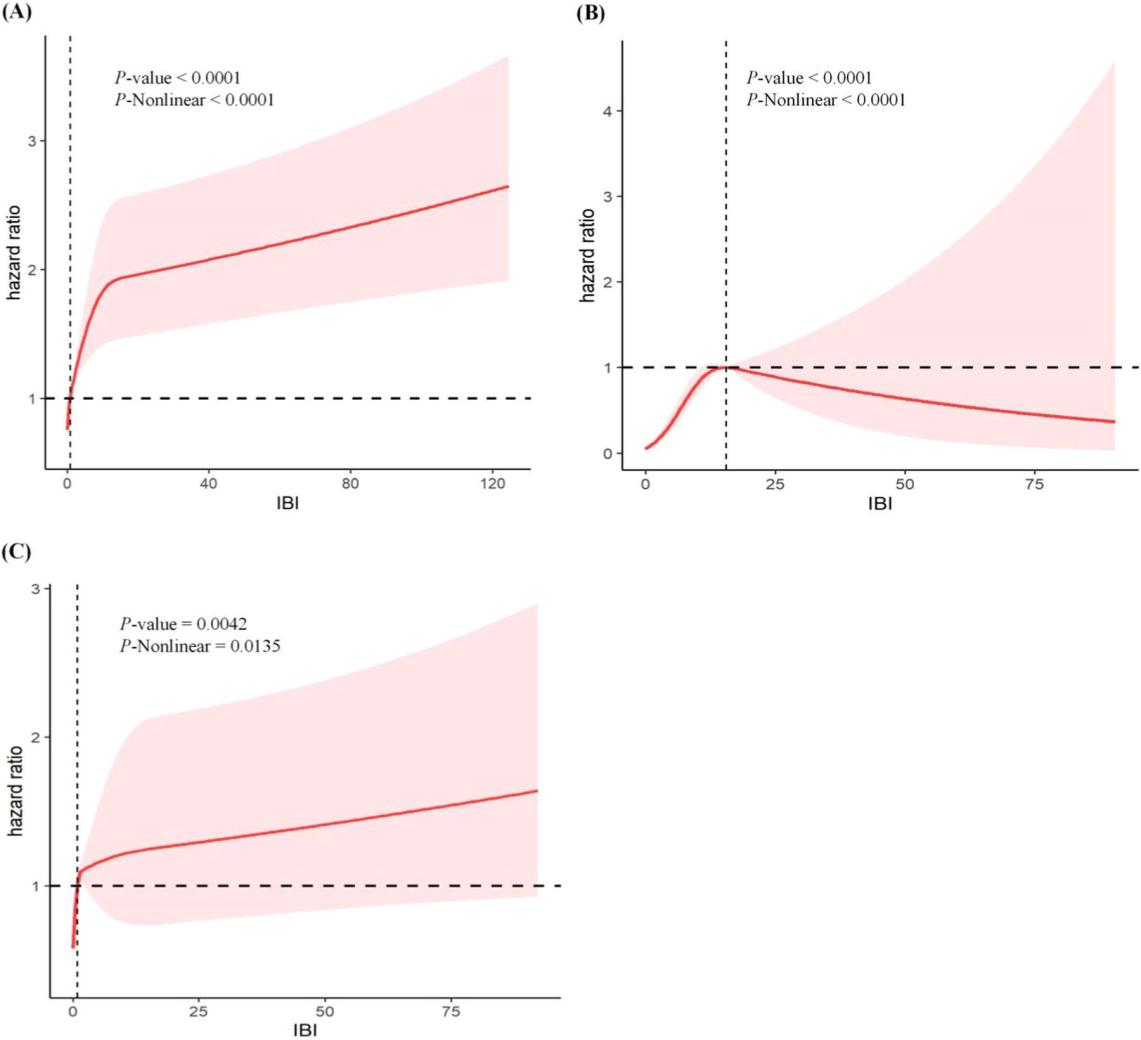
Restricted cubic spline analysis of HRs and 95% CIs illustrating the association between IBI scores and (A) all‐cause mortality, (B) CVD mortality, and (C) cancer‐specific mortality in cancer patients.

To elucidate the specific connection between IBI and HR, a restricted cubic spline regression analysis was conducted (Figure 3). After adjusting for multiple potential confounders, the nonlinear associations between IBI and all‐cause, CVD, and cancer‐specific mortality were statistically significant (*p* for nonlinearity < 0.05), highlighting the importance of using RCS to fit the Cox regression model for further evaluation.

### 4.5 Subgroup Analysis

#### 4.5.1 Stratified Analysis of IBI and Its Association With Mortality Outcomes

To evaluate the consistency of the IBI‐mortality association across diverse demographic and clinical subgroups, targeted analyses were performed considering factors like gender, ethnicity, education level, BMI, and conditions including hypertension, diabetes, hyperlipidemia, CKD, and CHD. The forest plot results (Table 3) indicate that there were no significant differences in the association with all‐cause mortality across subgroups such as gender, education level, marital status, BMI, diabetes, and CHD (*p* for interaction > 0.05).

**Table 3:** Subgroup and interaction analysis between IBI and mortality: (A) all‐cause mortality, (B) CVD mortality, and (C) cancer‐specific mortality. A weighted Cox regression model was employed for the subgroup analyses, with the forest plot also based on the weighted Cox regression model. Interaction analysis was performed using the Likelihood Ratio Test.

Characteristic	All‐cause mortality			CVD mortality			Cancer mortality		
	HR (95% CI)	*p* value	*p* for interaction	HR (95% CI)	*p* value	*p* for interaction	HR (95% CI)	*p* value	*p* for interaction
**Gender**			0.052			0.591			0.358
Female	1.01	< 0.0001		1.02	0.10		1.01	< 0.0001	
	(1.01, 1.01)			(1.00, 1.04)			(1.01, 1.01)		
Male	1.01	< 0.0001		1.01	< 0.0001		1.01	< 0.0001	
	(1.00, 1.01)			(1.01, 1.01)			(1.01, 1.01)		
**Ethnicity**			0.016			0.055			0.622
Mexican American	1.01	0.42		1.02	0.003		1.00	0.95	
	(0.99, 1.03)			(1.01, 1.04)			(0.96, 1.04)		
Non‐Hispanic White	1.01	< 0.0001		1.01	< 0.0001		1.01	< 0.0001	
	(1.01, 1.01)			(1.01, 1.01)			(1.01, 1.01)		
Non‐Hispanic Black	1.01	< 0.0001		1.02	0.03		1.01	0.34	
	(1.01, 1.02)			(1.00, 1.04)			(0.99, 1.03)		
Other	1.01	< 0.0001		0.44	< 0.0001		1.01	< 0.0001	
	(1.01, 1.01)			(0.31, 0.63)			(1.01, 1.01)		
**Education level**			0.769			0.283			0.463
High School or above	1.01	< 0.0001		1.01	< 0.0001		1.01	< 0.0001	
	(1.01, 1.01)			(1.01, 1.01)			(1.01, 1.01)		
Below high school	1.01	< 0.0001		1.03	0.02		1.01	< 0.0001	
	(1.01, 1.01)			(1.00, 1.05)			(1.01, 1.01)		
**Marital status**			0.052			0.050			0.362
Married/cohabitant	1.01	< 0.0001		1.02	< 0.0001		1.01	< 0.0001	
	(1.01, 1.01)			(1.01, 1.03)			(1.01, 1.01)		
Divorced/separated	1.00	< 0.0001		1.01	< 0.0001		1.01	< 0.0001	
	(1.00, 1.01)			(1.01, 1.01)			(1.01, 1.01)		
Widowed	1.01	< 0.0001		1.03	0.21		1.03	0.11	
	(1.00, 1.01)			(0.98, 1.09)			(0.99, 1.07)		
Never married	1.02	0.08		0.99	0.88		1.03	0.01	
	(1.00, 1.05)			(0.91, 1.08)			(1.01, 1.05)		
**Drinking status**			0.035			0.069			0.561
Former	1.01	< 0.0001		1.01	< 0.0001		1.01	0.18	
	(1.00, 1.01)			(1.00, 1.01)			(0.99, 1.03)		
Mild	1.01	< 0.0001		1.02	< 0.0001		1.01	< 0.0001	
	(1.01, 1.01)			(1.01, 1.03)			(1.01, 1.01)		
Moderate	1.04	0.07		1.06	0.01		1.04	0.23	
	(1.00, 1.08)			(1.02, 1.10)			(0.98, 1.11)		
Heavy	1.06	0.004		1.06	0.10		1.06	0.05	
	(1.02, 1.10)			(0.99, 1.14)			(1.00, 1.11)		
Never	1.01	< 0.0001		0.99	0.87		1.01	< 0.0001	
	(1.01, 1.01)			(0.84, 1.16)			(1.01, 1.01)		
**Smoking status**			0.017			0.069			0.714
Former	1.01	< 0.0001		1.02	0.04		1.01	< 0.0001	
	(1.01, 1.01)			(1.00, 1.03)			(1.01, 1.01)		
Now	1.01	< 0.0001		1.01	< 0.0001		1.02	0.06	
	(1.01, 1.01)			(1.01, 1.01)			(1.00, 1.04)		
Never	1.01	< 0.0001		1.02	< 0.0001		1.01	< 0.0001	
	(1.00, 1.01)			(1.01, 1.03)			(1.01, 1.01)		
**BMI**			0.105			< 0.001			0.013
< 25.0	1.01	< 0.0001		1.01	< 0.0001		1.05	< 0.0001	
	(1.00, 1.01)			(1.01, 1.01)			(1.03, 1.06)		
25–29.9	1.01	< 0.0001		1.03	< 0.0001		1.01	< 0.0001	
	(1.01, 1.01)			(1.02, 1.03)			(1.01, 1.01)		
≥ 30	1.01	< 0.0001		1.01	0.37		1.01	< 0.001	
	(1.01, 1.01)			(0.99, 1.02)			(1.00, 1.01)		
**Hypertension**			0.044			0.393			0.156
No	1.02	0.002		1.01	0.10		1.02	0.01	
	(1.01, 1.03)			(1.00, 1.03)			(1.00, 1.04)		
Yes	1.01	< 0.0001		1.01	< 0.0001		1.01	< 0.0001	
	(1.00, 1.01)			(1.01, 1.01)			(1.01, 1.01)		
**Diabetes**			0.113			0.097			0.251
DM	1.01	< 0.0001		1.01	< 0.0001		1.01	< 0.0001	
	(1.00, 1.01)			(1.00, 1.01)			(1.01, 1.01)		
IFG	1.01	< 0.0001		1.10	< 0.0001		1.05	0.01	
	(1.01, 1.01)			(1.05, 1.15)			(1.02, 1.09)		
IGT	1.08	0.1		0.97	0.78		1.06	0.78	
	(0.99, 1.18)			(0.79, 1.19)			(0.69, 1.64)		
No	1.02	< 0.0001		1.02	0.02		1.01	0.06	
	(1.01, 1.03)			(1.00, 1.03)			(1.00, 1.03)		
**Hyperlipidemia**			0.041			< 0.001			0.385
No	1.01	< 0.0001		1.02	< 0.0001		1.01	< 0.0001	
	(1.01, 1.01)			(1.02, 1.03)			(1.01, 1.01)		
Yes	1.01	< 0.0001		1.01	< 0.0001		1.01	< 0.0001	
	(1.00, 1.01)			(1.01, 1.01)			(1.01, 1.01)		
**CKD**			< 0.001			< 0.001			0.284
No	1.02	< 0.0001		1.03	< 0.0001		1.02	0.02	
	(1.01, 1.02)			(1.02, 1.03)			(1.00, 1.03)		
Yes	1.01	< 0.0001		1.01	< 0.0001		1.01	< 0.0001	
	(1.00, 1.01)			(1.00, 1.01)			(1.00, 1.01)		
**CHD**			0.619			0.418			0.007
No	1.01	< 0.0001		1.01	< 0.0001		1.01	< 0.0001	
	(1.00, 1.01)			(1.01, 1.01)			(1.01, 1.01)		
Yes	1.01	< 0.001		1.01	0.02		1.00	0.73	
	(1.00, 1.01)			(1.00, 1.02)			(0.99, 1.01)		

However, significant interactions were observed with ethnicity, drinking status, hypertension, hyperlipidemia, and CKD (*p* for interaction < 0.05). In the case of CVD mortality, similar nonsignificant trends were identified across gender, ethnicity, education level, drinking status, smoking status, hypertension diabetes, and CHD (*p* for interaction > 0.05). Notable differences were evident in marital status, BMI, hyperlipidemia, and CKD. Subgroup analyses revealed significant interactions between IBI and BMI or CHD in cancer‐specific mortality (*p* for interaction < 0.05).

### 4.6 The Predictive Ability of IBI for All‐Cause, CVD, and Cancer‐Related Mortality in Cancer Patients

**Figure 4:**
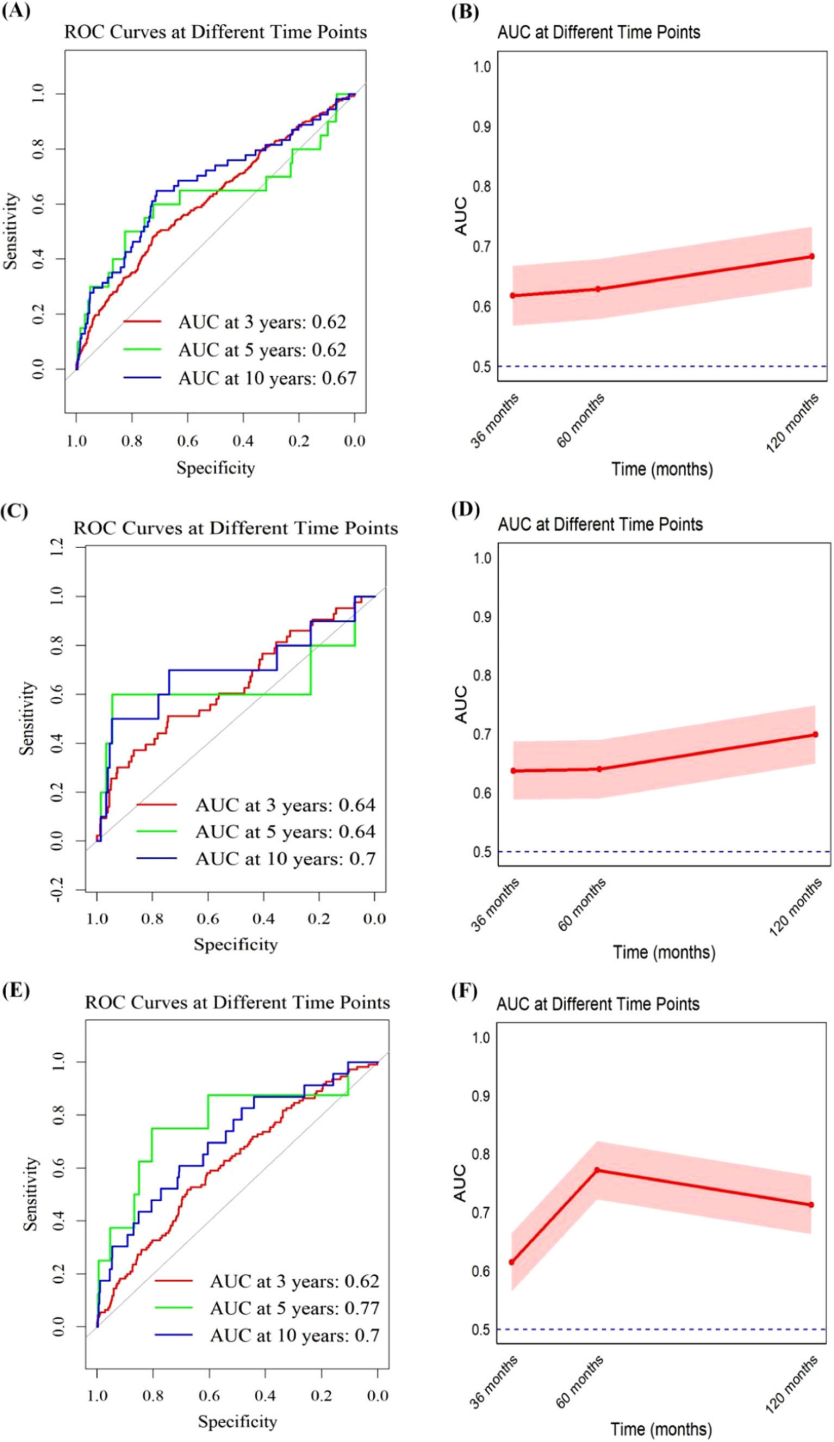
Time‐dependent ROC curves and corresponding AUC values (with 95% confidence intervals) for IBI in predicting (A, B) all‐cause mortality, (C, D) CVD mortality, and (E, F) cancer‐specific mortality.

The construction of the receiver operating characteristic (ROC), depicted in Figure 4, is crucial for assessing the diagnostic precision of the IBI for prognostic applications. Time‐dependent ROC analysis shows that the predictive capability of the IBI for all‐cause mortality reaches an area under the curve (AUC) of 0.62 at both 3 and 5 years, and 0.67 at 10 years, as illustrated in Figure 4A,B. Similarly, the AUC for the IBI in predicting mortality from CVD registers at 0.64 for both 3 and 5 years, and 0.70 at 10 years, as indicated in Figure 4C,D. Furthermore, the AUC values for the IBI in predicting cancer‐specific mortality are 0.62, 0.77, and 0.70 for 3, 5, and 10 years, respectively, illustrating the IBI's consistent predictive strength across different mortality outcomes over varying time periods.

## 5 Discussion

This study utilized multiple methods to comprehensively analyze the correlation between cancer patients and all‐cause mortality as well as cardiovascular mortality. By analyzing the examination data of 2748 cancer patients in NHANES, we found a relationship between IBI and all‐cause mortality, cardiovascular mortality, and cancer mortality. Using a weighted Cox regression model, we further revealed a nonlinear relationship between IBI and these mortality indices, which may indicate that the predictive power for mortality risk varies at different levels of inflammation. Analysis using Kaplan–Meier survival curves showed that patients with high IBI experienced poorer survival outcomes compared to those with low IBI. Additionally, RCS analysis demonstrated a nonlinear association between IBI and various mortality outcomes, including all‐cause, cardiovascular, and cancer‐related deaths among cancer patients. Significantly, time‐dependent ROC curve analysis demonstrated that the IBI's capacity to predict all‐cause mortality, as reflected by consistent AUC values at various time intervals (3, 5, and 10 years), underscores its utility as a dependable marker of inflammatory status in evaluating clinical prognoses.

These findings indicate that IBI can serve as a valuable adjunct to pathological staging in evaluating cancer patient prognoses. Previous research has extensively examined the correlation between IBI and survival rates in cancer patients [11]. Initially introduced by Xie, the IBI serves as a tool for assessing the inflammatory burden across various cancers and forecasting patient outcomes [12]. Their study revealed that the IBI not only distinguishes between prognoses based on varying levels of inflammation but also offers critical prognostic stratification across a broad spectrum of cancer patients. Subsequent research has methodically and extensively evaluated the predictive efficacy of different combinations of serum inflammatory markers in forecasting colorectal cancer outcomes. In these evaluations, the IBI demonstrated the greatest predictive accuracy [13].

An increasing body of evidence supports the notion that systemic inflammatory biomarkers in the blood are effective predictors of cancer prognosis across multiple types [14, 15]. Key characteristics of systemic inflammation are characterized by increased levels of circulating neutrophils, platelets, and CRP, accompanied by decreased levels of circulating lymphocytes and albumin [16]. The IBI, a biomarker that reflects bodily inflammation levels, is linked to heightened mortality risks via diverse mechanisms. IBI has proven invaluable for evaluating the intricacies of the inflammatory response, integrating three critical parameters: CRP [17], neutrophils, and lymphocytes [18]. Recent studies, such as the one by Modica et al., have further demonstrated the potential of systemic inflammatory biomarkers like NLR, PLR, and SII as valuable predictors of cancer outcomes, specifically in patients with sporadic medullary thyroid cancer [19]. Elevated serum CRP is considered the foremost clinical indicator of acute inflammation, whereas neutrophils and lymphocytes play crucial roles as cellular elements of the immune response. Concurrently, research indicates that CRP directly impairs the functionality of immune‐active cells, diminishing the body's cytotoxic immune response and consequently facilitating tumor immune escape [20]. This weakening of the immune defense and the tumor cell evasion mechanisms can contribute to the failure of cancer therapies, ultimately raising mortality rates [21]. Thus, current research supports the view that the IBI may serve as a superior predictor of outcomes for patients with various malignant tumors. This is because IBI effectively integrates and measures the interplay between acute and immune‐mediated inflammation, providing a balanced assessment of both inflammation types [22]. Furthermore, a high IBI independently correlates with significant risk factors, including compromised physical condition, malnutrition, cachexia, and an adverse short‐term prognosis [23, 24].

The interaction intensity between various cancers and their hosts differs, leading to diverse inflammatory burdens among cancer patients. The identification of links between the IBI and both cardiovascular and cancer mortality offers potential targets for clinical interventions. Given inflammation's critical role in the progression of cardiovascular diseases and cancers, regular monitoring of IBI could assist physicians in pinpointing high‐risk individuals [25]. This early identification facilitates timely interventions, which may significantly enhance patient outcomes via anti‐inflammatory therapies or other pertinent treatment approaches.

Our research possesses several significant advantages. Initially, the varied analytical techniques and prolonged follow‐up period enhanced the robustness of our findings. Furthermore, while the sample size might appear modest, the participants accurately reflect diverse social strata in the U.S. between 1999 and 2018, thereby ensuring our findings are widely applicable and representative. Although this study presents convincing evidence of the application value of IBI among cancer patients, it also has certain limitations. For instance, systemic inflammatory biomarkers were assessed at a single time point, which may vary over time. Given that the data encompass only US adult cancer patients, extrapolating the findings to individuals in other countries with different genetic backgrounds, diets, and lifestyles might be challenging. Furthermore, although we have attempted to account for many confounding factors, the possibility of omitting other significant covariates still exists. In addition, this study focused primarily on the association between IBI and mortality in cancer patients and failed to make direct comparisons with other commonly used single inflammatory markers (C‐reactive protein, white blood cell count, etc.). This limits our full understanding of the unique strengths and limitations of IBI relative to other inflammatory markers. Future studies should incorporate comparative analyses of IBI with other inflammatory markers to fully assess its utility and superiority in the clinical setting. Lastly, the findings of this study are based only on the NHANES database, and despite the broad representativeness of its data, validation in other independent databases is needed to ensure the robustness and generalizability of the results. Future studies should be validated in different datasets to further confirm the validity and reliability of IBI as a prognostic assessment tool for cancer.

## 6 Conclusion

In summary, IBI, as a novel indicator of systemic inflammation, can assess the inflammatory load across various cancer types, providing a promising and viable method for predicting outcomes in cancer patients. Its application in clinical prognostic assessments holds significant clinical value. With continued research and application, IBI is anticipated to become a vital tool in cancer management.

## Author Contributions

X.X.Q. made significant contributions to the idea and planning of the research, the writing of the paper, and the critical editing of the main ideas. Y.Y.Z., Y.J.Z., and M.Y. conducted the data acquisition, which involved collecting and analyzing clinical data. L.T. was involved in the development and planning of the research and provided feedback on the manuscript. Each author approved of the version of the manuscript that was submitted for publication and made substantial contributions to it.

## Ethics Statement

The survey was approved by the National Center for Health Statistics Ethics Review Board and in accordance with the Helsinki Declaration. The ethical approval numbers: NHANES 1999‐2004 (#98‐12), NHANES 2005‐2010 (#2005‐06), and NHANES 2011‐2018 (#2011‐17). Written informed consent was provided by each person to take part in this research. More details can be found at: https://www.cdc.gov/nchs/nhanes/irba98.htm.

## Conflicts of Interest

The authors declare no conflict of interest.

## Data Availability

This study analyzed publicly available datasets. The data are accessible at the following location: https://www.cdc.gov/nchs/nhanes/.
